# Supplementary material for: A novel incomplete hesitant fuzzy information supplement and clustering method for large-scale group decision-making
Source: PeerJ Comput Sci. 2024 Jan 16;10:e1803. doi: 10.7717/peerj-cs.1803 (PMC10807752; doi:10.7717/peerj-cs.1803)
Supplement: Supplemental Information 1 [file peerj-cs-10-1803-s001.docx]

**Appendix 1 Linguistic decision information of 20 DMs**

Linguistic decision information of DMs(e1-e5)

|  | c1 | c2 | c3 | c1 | c2 | c3 | c1 | c2 | c3 | c1 | c2 | c3 | c1 | c2 | c3 |
| --- | --- | --- | --- | --- | --- | --- | --- | --- | --- | --- | --- | --- | --- | --- | --- |
| e1-e5 | MH | $\varphi$ | ML | MH | $\varphi$ | $\varphi$ | MH | $\varphi$ | ML | ML | $\varphi$ | H | M | M | MH |
|  | M | MH | $\varphi$ | M | ML | M | MH | $\varphi$ | M | $\varphi$ | M | $\varphi$ | MH | M | MH |
|  | MH | $\varphi$ | M | MH | ML | $\varphi$ | M | H | MH | M | M | H | MH | H | M |
|  | H | M | MH | H | MH | M | H | ML | M | M | MH | M | MH | MH | ML |

Linguistic decision information of DMs(e6-e10)

|  | c1 | c2 | c3 | c1 | c2 | c3 | c1 | c2 | c3 | c1 | c2 | c3 | c1 | c2 | c3 |
| --- | --- | --- | --- | --- | --- | --- | --- | --- | --- | --- | --- | --- | --- | --- | --- |
| e6-e10 | M | MH | ML | MH | ML | $\varphi$ | MH | H | M | M | MH | MH | M | MH | M |
|  | M | $\varphi$ | $\varphi$ | M | $\varphi$ | M | MH | MH | M | M | $\varphi$ | MH | MH | L | M |
|  | MH | ML | M | MH | ML | MH | M | L | MH | M | $\varphi$ | H | H | M | M |
|  | H | M | MH | MH | M | M | H | MH | M | H | MH | M | MH | MH | ML |

Linguistic decision information of DMs(e11-e15)

|  | c1 | c2 | c3 | c1 | c2 | c3 | c1 | c2 | c3 | c1 | c2 | c3 | c1 | c2 | c3 |
| --- | --- | --- | --- | --- | --- | --- | --- | --- | --- | --- | --- | --- | --- | --- | --- |
| e11-e15 | M | MH | ML | MH | ML | M | MH | M | ML | ML | MH | M | MH | ML | M |
|  | MH | MH | H | M | H | M | MH | $\varphi$ | $\varphi$ | MH | MH | H | M | MH | M |
|  | MH | VH | M | MH | H | H | M | $\varphi$ | MH | ML | MH | H | MH | ML | H |
|  | M | MH | MH | H | MH | M | H | M | M | MH | M | M | H | M | M |

Linguistic decision information of DMs(e16-e20)

|  | c1 | c2 | c3 | c1 | c2 | c3 | c1 | c2 | c3 | c1 | c2 | c3 | c1 | c2 | c3 |
| --- | --- | --- | --- | --- | --- | --- | --- | --- | --- | --- | --- | --- | --- | --- | --- |
| e16-e20 | MH | MH | ML | ML | MH | H | M | M | MH | MH | ML | M | MH | MH | ML |
|  | MH | M | M | MH | M | MH | MH | L | M | M | MH | M | MH | M | M |
|  | M | H | MH | ML | M | H | H | MH | M | MH | ML | H | M | H | MH |
|  | H | ML | M | H | M | M | H | MH | ML | H | M | M | H | ML | M |
