# Supplementary material for: A novel incomplete hesitant fuzzy information supplement and clustering method for large-scale group decision-making
Source: PeerJ Comput Sci. 2024 Jan 16;10:e1803. doi: 10.7717/peerj-cs.1803 (PMC10807752; doi:10.7717/peerj-cs.1803)
Supplement: Supplemental Information 2 [file peerj-cs-10-1803-s002.docx]

**Appendix 2 Linguistic trust assessments among 20 DMs**

|  | e1 | e2 | e3 | e4 | e5 | e6 | e7 | e8 | e9 | e10 |
| --- | --- | --- | --- | --- | --- | --- | --- | --- | --- | --- |
| e1 | - |  |  |  |  | M | M | H | H |  |
| e2 |  | - |  |  | M |  |  |  |  |  |
| e3 |  |  | - |  |  | MH |  |  |  | M |
| e4 |  |  |  | - |  |  | MH | MH |  |  |
| e5 | ML |  | MH |  | - |  |  | M |  | M |
| e6 |  |  |  |  | M | - |  |  | MH |  |
| e7 |  |  |  | M |  |  | - | M |  |  |
| e8 | -M |  |  | MH |  |  |  | - |  |  |
| e9 |  |  |  |  |  |  |  |  | - | MH |
| e10 |  |  |  | MH |  |  |  |  |  | - |
| e11 |  |  | H |  |  |  | H |  |  |  |
| e12 |  | M |  |  |  |  |  |  |  |  |
| e13 |  | MH |  | H |  |  |  |  |  |  |
| e14 |  | M |  | H |  | M |  |  |  |  |
| e15 |  |  |  | -H |  |  |  |  |  | -MH |
| e16 | H |  |  |  |  |  |  | MH |  |  |
| e17 |  |  |  |  |  |  |  | H |  |  |
| e18 |  |  | M |  |  |  |  | H |  | M |
| e19 |  |  | MH |  |  |  |  |  |  |  |
| e20 |  |  | M |  |  |  |  |  |  |  |

|  | e11 | e12 | e13 | e14 | e15 | e16 | e17 | e18 | e19 | e20 |
| --- | --- | --- | --- | --- | --- | --- | --- | --- | --- | --- |
| e1 |  |  |  |  | -M |  |  |  | -MH |  |
| e2 |  | M | M |  |  |  |  |  |  |  |
| e3 | M |  |  | H |  |  |  |  |  |  |
| e4 |  |  | MH |  |  | H | M | M |  |  |
| e5 |  |  |  |  |  |  |  |  | H |  |
| e6 |  |  |  |  | MH |  |  | MH |  |  |
| e7 |  |  |  |  |  |  |  |  |  |  |
| e8 |  |  |  | -L |  |  |  |  |  | MH |
| e9 | MH |  |  |  |  |  |  |  |  |  |
| e10 | MH |  |  |  |  |  |  |  |  |  |
| e11 | - |  |  |  |  |  |  |  |  |  |
| e12 |  | - |  | ML | MH |  |  |  | H | H |
| e13 |  |  | - |  |  | ML | MH |  |  |  |
| e14 | M | M |  | - |  |  |  |  |  |  |
| e15 |  |  |  |  | - |  |  |  | -L |  |
| e16 |  |  |  |  | M | - |  |  |  |  |
| e17 |  |  |  |  |  |  | - |  |  |  |
| e18 |  |  |  |  |  |  |  | - |  |  |
| e19 |  |  |  |  |  |  |  |  | - |  |
| e20 |  | MH |  |  |  |  |  |  | M | - |
